# Spatially dependent Raman gain by vortex beam in a four-level *N*-typed atomic system

**DOI:** 10.1038/s41598-025-93083-5

**Published:** 2025-03-13

**Authors:** Tong Zhang, Kai-Kai Zhang, Xu Deng, Tao Shui, Wen-Xing Yang

**Affiliations:** 1 School of Physics and Optoelectronic Engineering, Yangtze University, Jingzhou, 434023 Hubei China; 2 School of Electronic Information and Electrical Engineering, Yangtze University, Jingzhou, 434023 Hubei China

**Keywords:** Optical physics, Quantum physics

## Abstract

An efficient scheme for controlling the spatial Raman gain is proposed in a cold atomic ensemble with a four-level *N*-typed configuration. The cold atoms are driven by the Laguerre-Gaussian (LG) vortex beams. Considering the control and driving fields are vortex beams and using experimentally achievable parameters, we identify the condition under which the topological charges (TCs) allows us to manipulate the radial distribution of the Raman gain spectra. It is found that the spatial Raman gain can be effectively controlled via adjusting the relevant optical parameters. Furthermore, we show interesting optical properties of vortex-induced transparency(VIT) and vortex-induced gain (VIG) via controlling the spatial Raman-gain spectrum. Finally we show that the azimuthal modulation of the Raman gain profiles can be realized and controlled when the control field consists of a travelling-wave (TW) field and two optical vortex fields. Our scheme may provide a feasible approach for constructing novel vortex beams based on cold atomic ensemble.

## Introduction

In the past few decades, the study of optical vortices has become a research topic of the fields of optics in recent years due to its potential applications in information transmission^1^, optical communication^2,3^, optical spanner^4,5^ and space exploration^6^. Note that a vortex beam has the characteristics of a helical phase and each photon carries an orbital angular momentum(OAM) of $l\hbar$, where *l*the topological charge (TC)^7^. The vortex beams can be divided into Lagure-Gaussian (LG) beam^8^, Bessel-Gaussian (BG) beam^9^, higher-order Bessel beam^10^and perfect optical vortex (POV) beam^11^. The above vortex beams can be generated via using different approaches, such as laser resonator^12^, spiral phase plate^13^, computational holography^14^, fiber-coupled converter^15–17^, photonic crystal converter^18^, polymer-based phase plate^19^, single-layer dielectric metasurface^20^, spatial light modulator^21,22^, strongly scattering media^23^, etc. In addition, the coloured vortex beam has also been realized via incoherent white light illumination^24^.

On the other hand, based on electromagnetically induced transparency(EIT)^25,26^, a cold atomic ensemble provides an effective platform for exploring spatially dependent light-matter interaction. Up to now, many interesting results have been achieved such as atom localizations^27–30^, electromagnetically induced gratings^31–33^, controllable photonic band gaps^34,35^, non-Hermitian systems^36–38^, ultraprecise Rydberg atomic localization^39^and Ferris wheel patterning^40^. In 2015, Alexander *et al.*proposed a technique to distinguish nonlinear processes by transferring the topological charge from the orbital angular momentum of laser radiation to coherent and directional optical fields^41^. The scheme by using a vortex beam to replace the optical wave has provided new possibilities for scientific research and technological applications in quantum information^42–45^. Since then, vortex light-atom interaction in cold atomic systems has led to many interesting quantum optical phenomena, such as vortex wave mixing^46–56^. Up to now, a host of breakthroughs have been made such as helical phase modulation^48–51^ and OAM transfer^52–57^. Moreover, the absorption of vortex beams in cold atomic systems has been extensively studied^58–63^. Hamid et al. suggest another scenario for the detection of the structured light by measuring the absorption profile of a weak nonvortex probe beam in a highly resonant five-level combined tripod and $\Lambda$(CTL) atom-light coupling setup^58^. In 2021, Dai et al. provided a way to observe the unique transport properties of the dynamic control of the vortex beam in a multilevel system^59^. Peng et al. proposed a scheme to adjust the spatial distribution of the refractive index using real parts of susceptibility in a four-level dense atomic ensemble^60^. As far as we know, no reports have been proposed for the manipulation of spatially dependent Raman gain.

In this paper, we investigate the properties of spatially dependent Raman gain and interesting phenomenon of vortex-induced transparency(VIT) and Raman gain(VIRG) in a cold atomic ensemble with a four-level *N*-typed configuration. This atomic system has been used to study atom localization^27,28^ and PT-antisymmetric system^33,38^. In comparison with references^59–61^, we use the control and driving fields with optical vortices to create the spatially distribution of Raman gain. Different from previous studies, the distinguishing features of this scheme are given as follows: first and foremost, we are interested in showing the tunability of spatial Raman gain, which are distinct from the scope of the optically controllable vortex-induced spatial absorption and spatial refractive index modulation in Refs^47,57–63^. Second, with the help of Raman scattering process, the TCs of the control and driving fields can be used to realize the transformation from spatial the transparency to the spatial Raman gain, thereby giving rise to different behavior of the probe gain in comparison with the results in Refs^61–63^. Third, by adjusting the detuning of the probe field and the intensity of the driving field, the spatial Raman gain can be drastically manipulated. Fourth, the azimuthally dependent Raman gain can be observed when the control field is a composite beam. our investigation offers distinct advantages in the generation of new structured beams owing to amplification compared with^58,61^. Our scheme may have potential applications optical communication.

## Models and equations

**Fig. 1:**
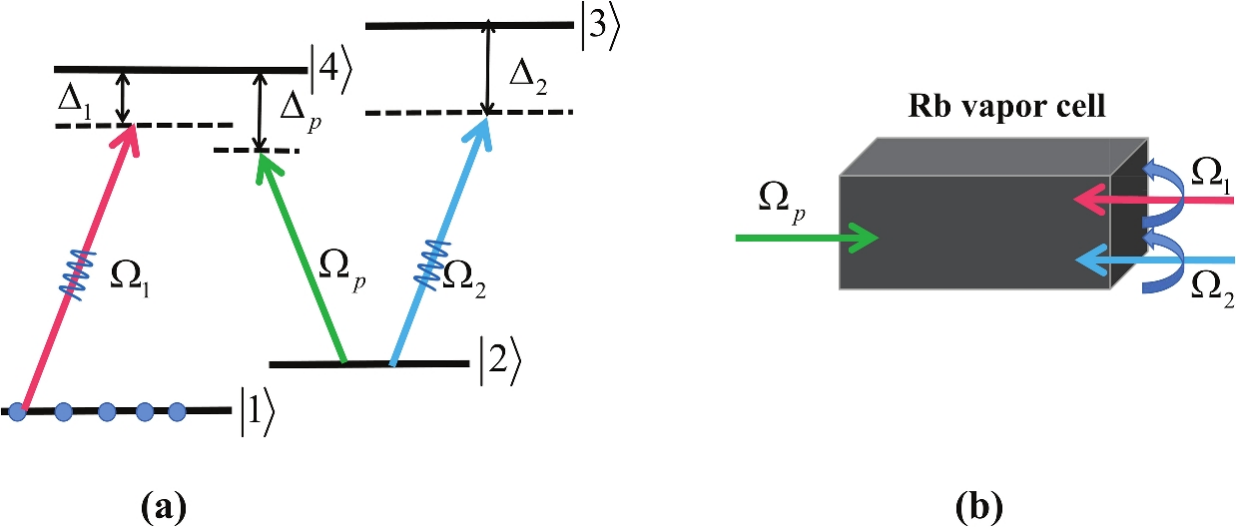
(**a**) (Color online) Schematic of four-level atomic system. Here $\Omega _1$ and $\Omega _2$ are the respective Rabi frequencies of pump control fields and $\Omega _p$ is the Rabi frequency of the weak probe field. (**b**) Possible arrangement of the experimental setup, which is composed of a cold rubidium (Rb) atomic vapor cell, a nonvortex probe beam, a vortex control field, and a vortex driving field.

As shown in Fig. 1, we consider a four-level atomic system with two excited states $\left| \mathrm{{3}} \right\rangle$, $\left| \mathrm{{4}} \right\rangle$ and two ground states $\left| \mathrm{{1}} \right\rangle$, $\left| \mathrm{{2}} \right\rangle$. The experimental system for this scheme is realized by cold $^{87}$Rb atoms^64^ with $|5^2S_{1/2}, F=1 \rangle$, $|5^2S_{1/2}, F=2 \rangle$, $|5^2P_{1/2}, F=1\rangle$, $|5^2P_{1/2}, F=2\rangle$ behaving $|1\rangle$, $|2\rangle$, $|3\rangle$ and $|4\rangle$, respectively. The transition $|4\rangle \rightarrow |2\rangle$ is driven by a weak probe field with Rabi frequency $\Omega _{p}=\mu _{42} E_p/\hbar$. A control field with Rabi frequency $\Omega _{1}=\mu _{41} E_c/\hbar$ is applied to the transition $|4\rangle \rightarrow |1\rangle$ and the transition $|3\rangle \rightarrow |2\rangle$ is driven by a strong driving field with Rabi frequency $\Omega _{2}=\mu _{32} E_c/\hbar$. Here, $\mu _{42}$, $\mu _{41}$ and $\mu _{32}$ are the corresponding electric-dipole matrix moments. Note that the applied field $\Omega _{j}$(*j*=1,2) is a LG vortex beam, which can be written as^59^:

1 $$\begin{aligned} \Omega _{j} = \Omega _{j0} (\frac{r}{\omega _0})^{\left| l_{j} \right| }e^{\left( -\frac{r^2}{\omega _0^2}\right) }e^{-il_{j}\theta } , \end{aligned}$$

where $\Omega _{j0}$ is the initial Rabi frequency of the corresponding vortex beam. *r* and $\theta$ indicate the radial position and the azimuthal angle, respectively. $\omega _0$ is the beam waist. $e^{-il_{j}\theta }$ is the helical phase factor with the optical OAM $\hbar l_j$ along the propagation axis.

Under the electric-dipole and rotating-wave approximations, the interaction Hamiltonian for the *N*-type atomic system is given by ($\hbar$=1)

2 $$\begin{aligned} \begin{array}{c} {H_I} = -(\Delta _p-\Delta _1)\left| 2 \right\rangle \left\langle 2 \right| -\Delta _p\left| 3 \right\rangle \left\langle 3 \right| -\Delta _2\left| 4 \right\rangle \left\langle 4 \right| - \frac{1 }{2}[{\Omega _1}\left| 4 \right\rangle \left\langle 1 \right| + {\Omega _2}\left| 3 \right\rangle \left\langle 2 \right| + {\Omega _p} \left| 4 \right\rangle \left\langle 2 \right| + H.c.], \end{array} \end{aligned}$$

where $\Delta _1= \omega _{41} - \omega _1$, $\Delta _2= \omega _{32} - \omega _2$, $\Delta _p= \omega _{42} - \omega _p$ are the detunings of the corresponding applied fields, respectively. The equations of motion for the density matrix elements can be written as

3 $$\begin{aligned} \dot{\rho _{11}}= & \frac{i}{2}(\Omega _1\rho _{41}-\Omega _1^*\rho _{14}^*)+\gamma _{41}\rho _{44} + \gamma _{31}\rho _{43},\end{aligned}$$

4 $$\begin{aligned} \dot{\rho _{33}}= & \frac{i}{2}(\Omega _2\rho _{23}-\Omega _2^*\rho _{32}^*)-(\gamma _{32} + \gamma _{31})\rho _{33},\end{aligned}$$

5 $$\begin{aligned} \dot{\rho _{44}}= & \frac{i}{2}(\Omega _1\rho _{14}+\Omega _p\rho _{24}-\Omega _1^*\rho _{41}^*-\Omega _p^*\rho _{42}^*)-(\gamma _{41} + \gamma _{42})\rho _{44},\end{aligned}$$

6 $$\begin{aligned} \dot{\rho _{21}}= & [(i\Delta _1-\Delta _p)-\Gamma _{21}]\rho _{21}+\frac{i}{2}\Omega _1\rho _{24}-\frac{i}{2}\Omega _p\rho _{14}, \end{aligned}$$

7 $$\begin{aligned} \dot{\rho _{31}}= & [(i(\Delta _p-\Delta _1-\Delta _2)-\Gamma _{31}]\rho _{31}+\frac{i}{2}(\Omega _2\rho _{21}-\Omega _p\rho _{34}),\end{aligned}$$

8 $$\begin{aligned} \dot{\rho _{32}}= & (-i\Delta _2-\Gamma _{32})\rho _{32}+\frac{i}{2}\Omega _2(\rho _{22}-\rho _{33})-\frac{i}{2}\Omega _p\rho _{34},\end{aligned}$$

9 $$\begin{aligned} \dot{\rho _{41}}= & -(i\Delta _1+\Gamma _{41})\rho _{41}+\frac{i}{2}\Omega _1(\rho _{11}-\rho _{44})+\frac{i}{2}\Omega _p\rho _{21},\end{aligned}$$

10 $$\begin{aligned} \dot{\rho _{42}}= & -(i\Delta _p+\Gamma _{42})\rho _{42} + \frac{i}{2}\Omega _p(\rho _{22}-\rho _{44})+\frac{i}{2}\Omega _1\rho _{12}-\frac{i}{2}\Omega _2\rho _{43},\end{aligned}$$

11 $$\begin{aligned} \dot{\rho _{43}}= & [i(\Delta _2-\Delta _p)-\Gamma _{43}]\rho _{43}+\frac{i}{2}(\Omega _1\rho _{21}+\Omega _p\rho _{23}-\Omega _2\rho _{42}), \end{aligned}$$

where $\gamma _{ij}$ (*i*, *j* = 1, 2, 3, 4; $i> j$) is the spontaneous emission decay rate from the state $\left| i \right\rangle$ to the state $\left| j \right\rangle$ and $\Gamma _{ij}$ is the decay rate of the coherence between the states $\left| i \right\rangle$ and $\left| j \right\rangle$.

In the limit of the weak probe field, i.e.,${\Omega _p} \ll {\Omega _1},{\Omega _2}$, the depletions of the ground states $\left| 1 \right\rangle$ from the initial values are negligible. Then, to he first order one has $\rho _{11}+\rho _{22}+\rho _{33}+\rho _{44}=1$. For the Raman gain process, we consider that the atom is initially prepared in the ground state $\left| 1 \right\rangle$^27,33,38^, i.e., $\rho _{11}=1$ and $\rho _{22}=\rho _{33}=\rho _{44}=0$. At the second order, we obtain

12 $$\begin{aligned} \rho _{42}&= -\frac{i \Omega _p \Omega _1^2 }{A} \Bigg \{ \frac{2 \Gamma _{41}[\Gamma _{43}-i(\Delta _p-\Delta _2)]}{(\gamma _{14}+\gamma _{24})(\Gamma _{41}^2+\Delta _1^2)}+\frac{B}{C} \Bigg \}, \end{aligned}$$

with

$$\begin{aligned} A= & (\Gamma _{42} - i\Delta _p)[\Gamma _{43} - i(\Delta _p - \Delta _2)] + \left| \Omega _2 \right| ^2/4, \\ B= & [{\Gamma _{43}} - i({\Delta _p} - {\Delta _2})][{\Gamma _{31}} - i({\Delta _p} - {\Delta _1} - {\Delta _2})]{\left| {{\Omega _2}} \right| ^2}/4, \\ C= & ({\Gamma _{41}} + i{\Delta _1})\{ [{\Gamma _{12}} - i({\Delta _p} - {\Delta _1})][{\Gamma _{31}} - i({\Delta _p} - {\Delta _1} - {\Delta _2})]+ {\left| {{\Omega _2}} \right| ^2}/4\}. \end{aligned}$$

The nonlinear optical response of the atomic system can be described by the nonlinear Raman susceptibility $\chi$, which can be written as

13 $$\begin{aligned} \chi = \frac{2N \left| \vec {u}_{42} \right| ^2}{\hbar \varepsilon _{0}\Omega _p}\rho _{42}, \end{aligned}$$

where *N* is the atom number density and $\varepsilon _{0}$ is dielectric constant in vacuum. Note that the imaginary part Im$(\chi )$ of the probe susceptibility $\chi$ represents the absorption and gain properties, which can be written as

14 $$\begin{aligned} \text {Im}(\chi )=-\frac{2N \left| \vec {u}_{42} \right| ^2\Omega _1^2}{\hbar \varepsilon _{0}} \left\{ \frac{\kappa _1(\kappa _2^2+\kappa _3^2)(\kappa _5\kappa _8+\kappa _4\kappa _9)+\Gamma _{41}(\kappa _8^2+\kappa _9^2)[\kappa _2\Gamma _{43}+\kappa _3(\Delta _p-\Delta _2)]}{\kappa _1(\kappa _2^2+\kappa _3^2)(\kappa _8^2+\kappa _9^2)} \right\} , \end{aligned}$$

in which $$\kappa _1 =(\gamma _{41} + \gamma _{42})(\Gamma _{41}^2 + \Delta _1^2)$$, $$\kappa _2 = \Gamma _{42}\Gamma _{43} - \Delta _p(\Delta _p - \Delta _2) + \Omega _2^2/4$$,$$\kappa _3 = \Gamma _{43}\Delta _p + \Gamma _{42}(\Delta _p - \Delta _2)$$, $$\kappa _4 = \Gamma _{31}(\Delta _2-\Delta _p)-\Gamma _{43}(\Delta _p-\Delta _1-\Delta _2)$$, $$\kappa _5 = \Gamma _{43}\Gamma _{31} + (\Delta _2 - \Delta _p)$$$$(\Delta _p - \Delta _1 - \Delta _2) - \Omega _2^2/4$$, $$\kappa _6 = \Gamma _{21} \Gamma _{31} - (\Delta _p - \Delta _1)(\Delta _p - \Delta _1 - \Delta _2) + \Omega _2^2/4$$, $$\kappa _7 = \Gamma _{31}(\Delta _p - \Delta _1) + \Gamma _{21}(\Delta _p - \Delta _1 - \Delta _2)$$, $$\kappa _8 = (\Gamma _{41}\kappa _6 + \Delta _1\kappa _7)\kappa _2$$$$- (\Gamma _{41}\kappa _7 - \Delta _1\kappa _6)\kappa _3$$, $$\kappa _9 = - (\Gamma _{41}\kappa _6 + \Delta _1\kappa _7)\kappa _3 - (\Gamma _{41}\kappa _7 - \Delta _1\kappa _6)\kappa _2$$. For the Raman gain process, we can define the gain coefficient $$\beta =-\text {Im}(\chi )$$. In the following, we use the standardized gain coefficient, i.e., $$\beta /\max (\beta )$$, to describe the characteristics of the Raman gain spectrum. For $$^{87}$$Rb D1 line, the parameters of the atom are taken to be $$\Gamma _{41} = \Gamma _{42} = \Gamma _{31} = \Gamma _{43} /2 = \gamma =\pi \times 5.75$$ MHz, $$\gamma _{41} = \gamma _{31} = \pi \times 5.75$$ MHz and $$\Gamma _{21} = 10$$KHz^33^.

## Results and discussions

**Control field and driving field are vortex beams**

**Fig. 2:**
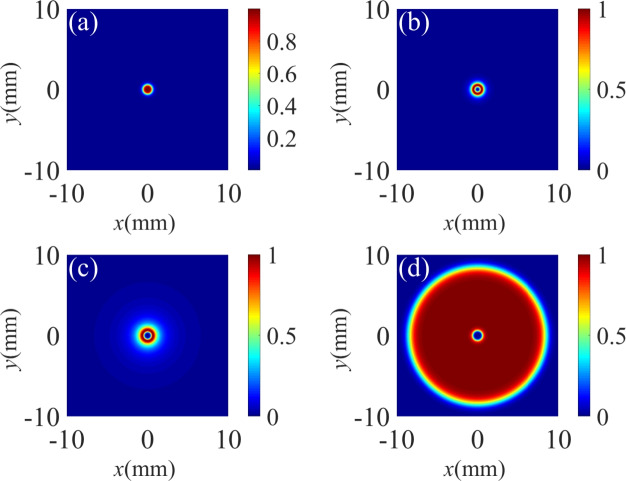
The standardized spatial Raman gain coefficient for different TCs of the control field. (**a**) $l_1$ = 0, (**b**) $l_1$ = 1, (**c**) $l_1$ = 2, (**d**) $l_1$ = 3. Other parameters are: ${\Delta _1} = 0$, ${\Delta _2} = 0$, ${\Delta _p} = 0$, $\omega _0=3$ mm. $\Omega _p= 0.01 \gamma$, $\Omega _{10} =10 \gamma$, $\Omega _{20}=6 \gamma$, $l_2$ = 3.

**Fig. 3:**
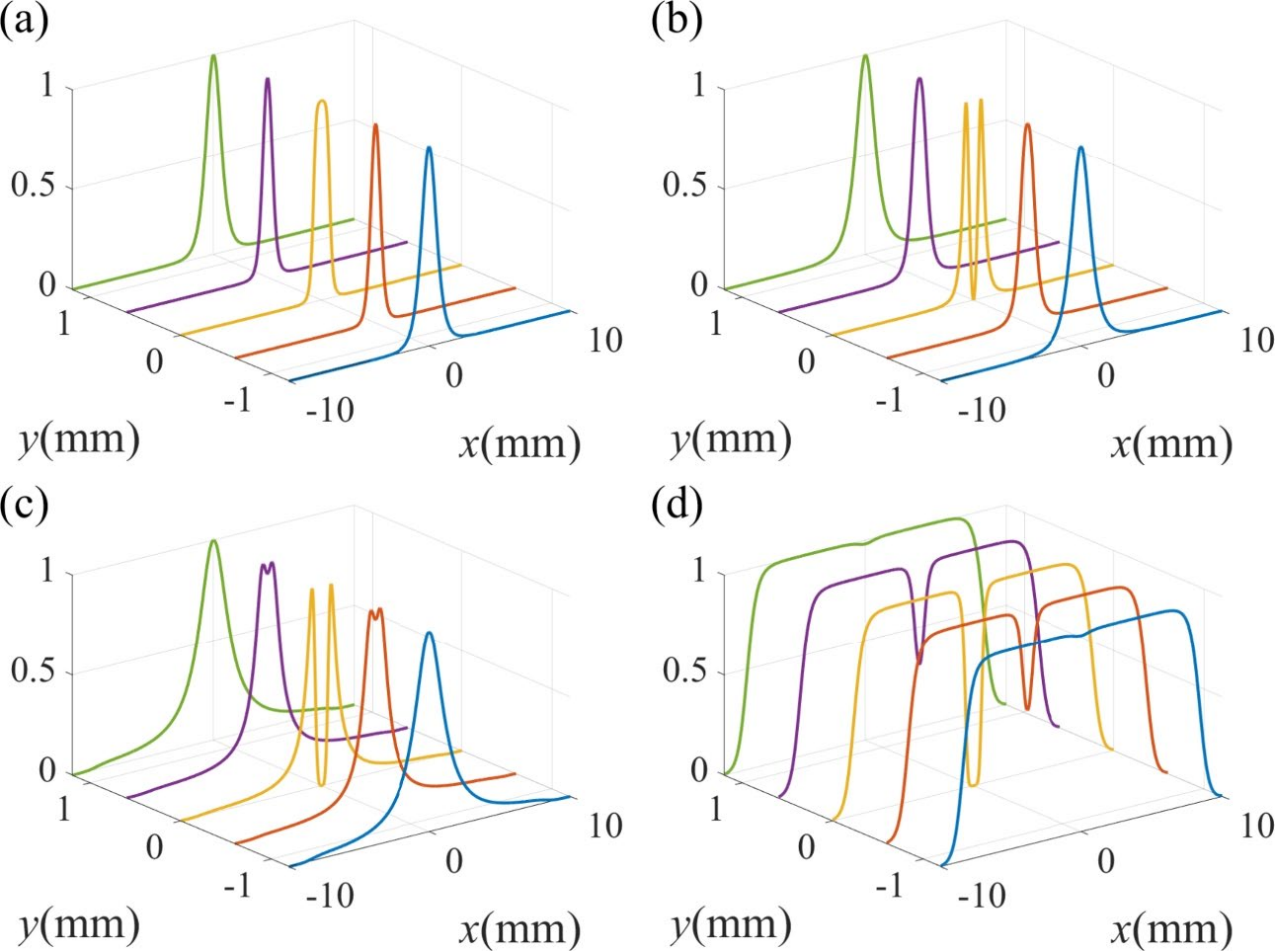
The standardized probe spatial Raman gain spectrum for different TCs. (**a**) $l_1$ = 0, (**b**) $l_1$ = 1, (**c**) $l_1$ = 2, (**d**) $l_1$ = 3. Other parameters are the same as in Fig. 2d.

**Fig. 4:**
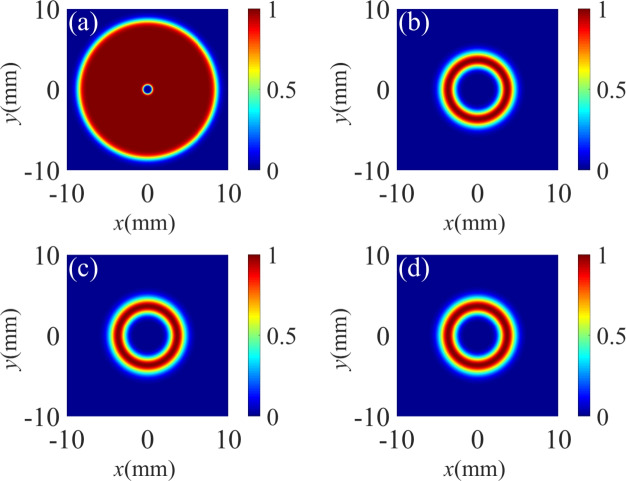
The standardized spatial Raman gain coefficient for different detunings $\Delta _p$ of probe field. (**a**) $\Delta _p = 1 \gamma$; (**b**) $\Delta _p = 3 \gamma$; (**c**) $\Delta _p = 5 \gamma$; (**d**) $\Delta _p = 7 \gamma$. The other parameters are the same as in Fig. 2d except for ${\Delta _1} = 1\gamma$, ${\Delta _2} = 1\gamma$.

**Fig. 5:**
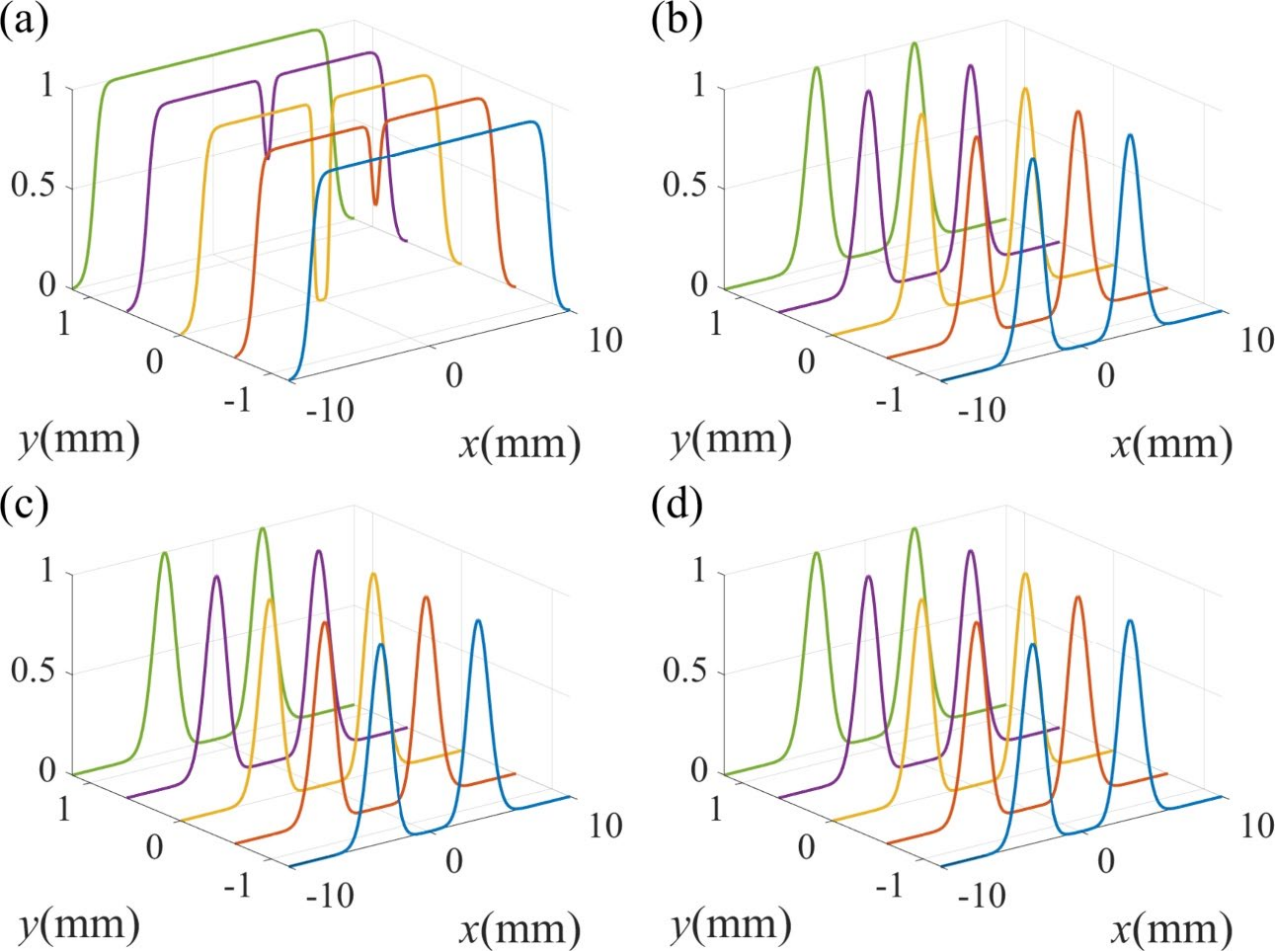
The standardized probe spatial Raman gain spectrum for different detunings $\Delta _p$ of probe field. (**a**) $\Delta _p = 1 \gamma$; (**b**) $\Delta _p = 3 \gamma$; (**c**) $\Delta _p = 5 \gamma$; (**d**) $\Delta _p = 7 \gamma$. The other parameters are the same as in Fig. 4.

We first investigate the influence of the TC of the vortex control field $\Omega _1$ on the Raman gain coefficient in Fig. 2. Here, the strong driving field is a vortex beam with the TC $l_2$ = 3. When the TC of the control field is $l_1$ = 0, meaning that the control field is a non-vortex beam, a Raman gain peak occurs at the vortex core of the driving field, which is surrounded by a transparent area (see Fig. 2a). As the TC of the control field is tuned to $l_1$ = 1, the gain area is transformed into a crater-like pattern of an annular structure, while the central region changes from a transition from vortex-induced gain (VIG) to vortex-induced transparency (VIT) in Fig. 2b. When the TC is $l_1$ = 2, it is found that the Raman gain exhibits a crater-like pattern, but the radius of the crater-like pattern increases (see Fig. 2c). As shown in Fig. 2d, when we set $l_1 = 3$, the Raman gain area becomes very wide. According to the above discussion, one can conclude that the spatial Raman gain is changed significantly with the increase of $l_1$ from 0 to 3.

We have demonstrated that the Raman gain profile is sensitively dependent on the TC of the vortex beams. In the following, we discuss in detail the effect of the vortex beams at several spectral lines. In Fig. 3, we show the normalized Raman gain at some specific locations. When the control field is a non-vortex beam, i.e., $l_1$ = 0, a sharp Raman-gain peak can be observed in the vicinity of $y = 0$ as illustrated in Fig. 3a. As the TC of the vortex driving field increases to $l_1$ = 1, as shown in Fig. 3b, the Raman gain peak at $y = 0$ splits into two peaks, resulting in the emergence of a transparent window at origin of the coordinate plane $(x, y) = (0, 0)$ due to the quantum destructive interference between the two steps $\left| 1 \right\rangle \leftrightarrow \left| 4 \right\rangle \leftrightarrow \left| 2 \right\rangle$ and $\left| 2 \right\rangle \leftrightarrow \left| 3 \right\rangle$^65^. Besides, there are four gain peaks which are symmetrical with respect to $x = 0$. In the case of $l_1$=2, as shown in Fig. 3c, the transparent window at the point of $(x, y) = (0, 0)$ becomes larger and the two adjacent gain peaks are sagged compared with the case of Fig. 3b. With the increase of $l_1$ = 3, the Raman gain peaks one both sides of $x = 0$ are flattened and broadened.

We then examine the influence of the detuning $\Delta _p$ of the probe field on the Raman gain coefficient in Fig. 4. When we adjust the probe detuning to $\Delta _p = \gamma$, the Raman gain spectrum appears as a massive crater shape of the toroidal structure with a small transparent window in the central region (see Fig. 4a). As the probe detuning is tuned to $\Delta _p = 3 \gamma$, the radius of the crater-like pattern of the spatial Raman gain decreases, while the transparent region at the center increases (see Fig. 4b). When the probe detuning increases to $\Delta _p = 5 \gamma$ and $\Delta _p = 7 \gamma$, the crater-like pattern of the spatial Raman gain is almost unchanged (see Fig. 4c,d).

In Fig. 5, we show the normalized Raman gain at some specific locations for different values of the probe detuning $\Delta _p$. When $\Delta _p = \gamma$, There exists two flat gain peaks at $y = 0$ with a small transparent window. When the spatial position moves away from $y = 0$, transparent window becomes weaken and even disappears (see Fig. 5a). Under the condition of $\Delta _p = 3 \gamma$, we can see two gain peaks and a wider transparent window in the spectrum (see Fig. 5b). As the detuning of the probe field is changed to $5 \gamma$ and $7 \gamma$, these spectra profiles with two gain peaks and one transparent window remain unchanged (see Fig. 5c,d). Therefore, one can conclude that the gain spectra lines are robust to larger probe detuning.

**Fig. 6:**
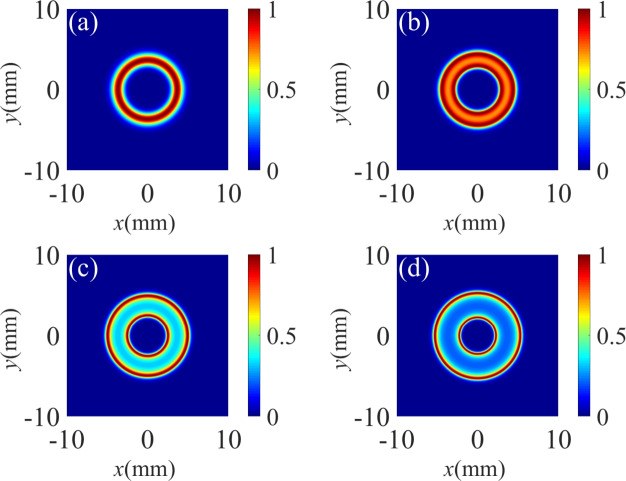
The standardized spatial Raman gain coefficient for different intensities of driving field $\Omega _{20}$. (**a**) $\Omega _{20}$ = 15 $\gamma$; (**b**)$\Omega _{20}$ = 20 $\gamma$; (**c**) $\Omega _{20}$ = 25 $\gamma$ (**d**) $\Omega _{20}$ = 30 $\gamma$. The other parameters are the same as in Fig. 4c.

We further explore the dependence of the Raman gain coefficient on the intensity $\Omega _{20}$ of the vortex driving field in Figs. 6. When $\Omega _{20} = 15 \gamma$, the Raman gain spectrum exhibits a crater-like pattern (see Fig. 6a). As $\Omega _{20}$ increases to $20 \gamma$, the Raman gain spectrum evolutes to a double-ring volcanic crater-like pattern (see Fig. 6b). When $\Omega _{20}$ increases to $25 \gamma$ and $30 \gamma$, as shown in Fig. 6c,d, the gain in the region between the two concentric rings continuously decreases and the distance between the two concentric rings becomes larger. The reason is that the increase of the intensity of the vortex driving field induces gain saturation at the position of $r=3.5$ mm, which would lead to the decrease of Raman gain.

**Fig. 7:**
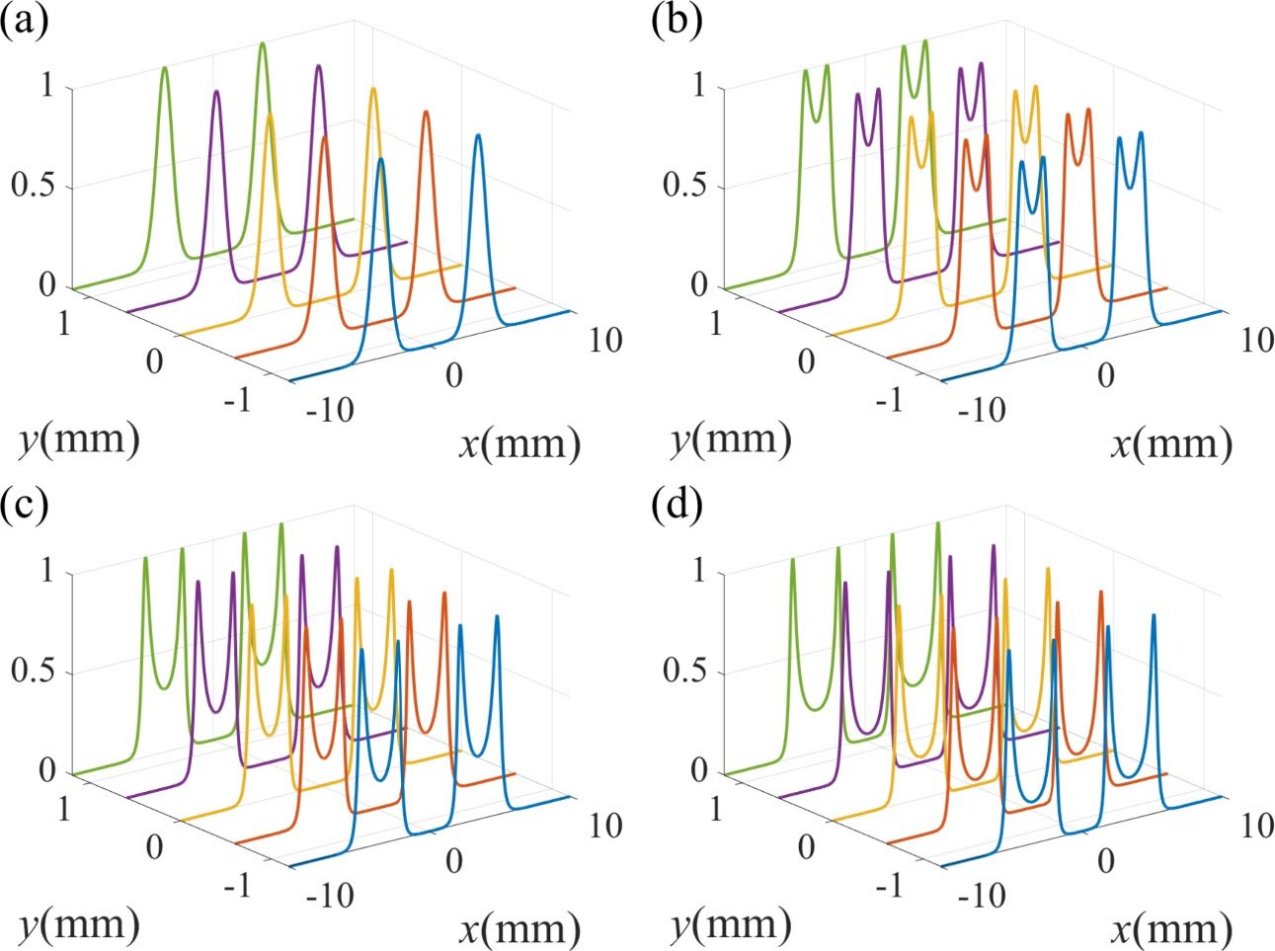
The standardized probe spatial Raman gain spectrum for different intensities of driving field $\Omega _{20}$. (**a**) $\Omega _{20}$ = 15 $\gamma$; (**b**) $\Omega _{20}$ = 20 $\gamma$; (**c**) $\Omega _{20}$ = 25 $\gamma$ (**d**) $\Omega _{20}$ = 30 $\gamma$. The other parameters are the same as in Fig. 4c.

In Fig. 7, we show the normalized Raman gain at some specific locations for different values of the driving intensity $\Omega _{20}$. When $\Omega _{20}$ is $15 \gamma$, there exists two shape gain peaks and a wide transparent window in the spectrum (see Fig. 7a). As the intensity of the driving field increases to $20 \gamma$, as shown in Fig. 7b, the two Raman gain peaks in the vicinity of $x = 0$ split. As $\Omega _{20}$ further increases to $25 \gamma$, one can obtain four gain peaks with the emergence of a transparent window and two weak-gain regions (see Fig. 7c). When $\Omega _{20}$ increases to $30 \gamma$, the transparent window is narrowed and the gain coefficient of the two weak-gain regions decreases (see Fig. 7d).

**Fig. 8:**
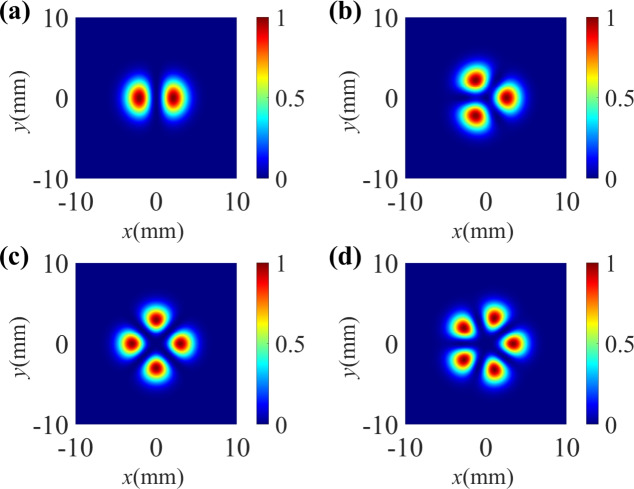
The standardized spatially dependent Raman gain profile of the probe beam for different TCs of control field. (**a**) $l_1 = 1, l_2= -1$; (**b**) $l_1 = 2, l_2= -1$; (**c**) $l_1 = 2, l_2= -2$; (**d**) $l_1 = 2, l_2= -3$. The other parameters are the same as in Fig. 2a,except for $\Omega _C = 0.5 \gamma$, $\Omega _C$ = 0, $\Omega _{c10} = \Omega _{c20} = 10 \gamma$.

**Fig. 9:**
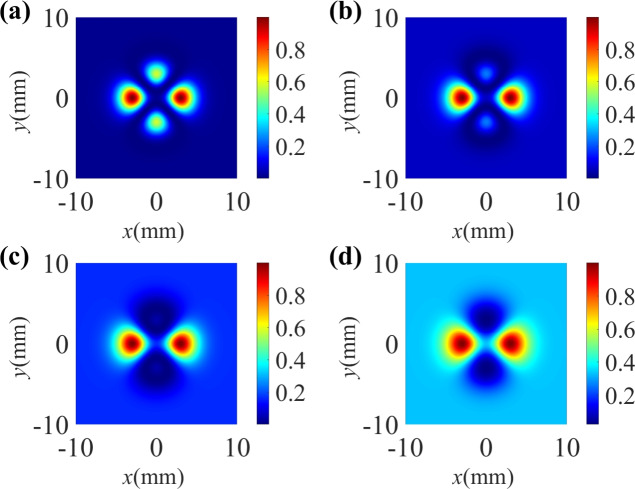
The standardized spatially dependent Raman gain profile of the probe beam for different Rabi frequencies $\Omega _C$. (**a**) $\Omega _C = 1 \gamma$, (**b**) $\Omega _C = 2.5 \gamma$, (**c**) $\Omega _C = 5 \gamma$, (**d**) $\Omega _C = 10 \gamma$. The other parameters are the same as in Fig. 8c.

0.2 Control field is a composite beam

In this section, we assume that the driving field is a travelling-wave (TW) field and the control field consists of a TW field and two optical vortex fields with the form^61^

15 $$\begin{aligned} \Omega _{1} = \Omega _C + \Omega _{c10} (\frac{r}{\omega _0})^{\left| l_1 \right| }e^{\left( -\frac{r^2}{\omega _0^2}\right) }e^{-il_1\theta }+ \Omega _{c20}(\frac{r}{\omega _0})^{\left| l_2 \right| }e^{\left( -\frac{r^2}{\omega _0^2}\right) }e^{-il_2\theta }, \end{aligned}$$

where $\Omega _C$, $\Omega _{c10}$ and $\Omega _{c20}$ are the intensities of the corresponding components of the control field. The coaxial interference between the three components of the control field can induce the azimuthal modulation of the Raman gain.

In Fig. 8, we investigate the influence of the TCs of the two vortex components on the Raman gain coefficient. The bright structures represent the positions of Raman gain, while the blue areas correspond to the regions of optical transparency. In the case of $l_1 = -l_2= 1$, the spatial Raman gain profile shows two bright spots with petal-like patterns (see Fig. 8a). When $l_1$ = 2 and $l_2$ = −1, as shown in Fig. 8b, the Raman gain exhibits three bright spots with the same size. When the TC $l_1$ is unchanged and $l_2$ increases from −1 to −3, the Raman gain display a -fold symmetry distributed bright spots in regions of spatial Raman gain (see Fig. 8c,d). Thus, we can clearly conclude that the number of bright spots in the Raman gain region is directly equal to $\left| l_1 - l_2 \right|$.

In Fig. 9, we analyze the influence of the intensity $\Omega _C$ of the TW component on the Raman gain coefficient. Obviously, the azimuthal distribution of the spatial Raman gain is sensitive to $\Omega _C$. For $\Omega _C$ = 1 $\gamma$ and $\Omega _C$ = 2.5 $\gamma$, the spatial distribution of the Raman gain shows four petal-like bright spots in the four different quadrants, half the number of the four spots have weakened energy (see Fig. 9a,b). With a further increase of $\Omega _C$ (i.e., $\Omega _C$ = 5 $\gamma$), the two weak bright spots almost completely disappear, which means two areas of optical transparency occurs (see Fig. 9c). As $\Omega _C$ increases to 10 $\gamma$, we can clearly observe two Raman gain areas and two optical transparency areas (see Fig. 9d). Therefore, the azimuthal distribution the Raman gain can be effectively manipulated via adjusting the intensity $\Omega _C$ of the TW field.

## Conclusions

In conclusion, we have theoretically investigated the spatially-dependent Raman gain in a four-level *N*-typed atomic system. In the presence of the vortex control and driving fields, the spatial distribution of the Raman gain spectra, such as VIT and VIG, can be controlled via adjusting the TCs of the optical vortices. It is demonstrated that the probe detuning and the driving intensity play different roles on the spatial Raman gain spectra. The gain spectral profiles are robust to the larger detuning of probe field and sensitive to the intensity of the driving field. Furthermore, we can observe the azimuthal distributed bright spots for the Raman gain profiles when the control field is the superposition of a travelling-wave (TW) field and two optical vortex fields. It is found the the azimuthal modulation of the Raman gain profiles can be manipulated via adjusting the TCS of the two vortex components and the intensity of the TW component. Our scheme may provide a feasible approach for constructing novel vortex beams based on cold atomic ensemble.

## Data Availability

All data generated or analysed during this study are included in this published article.
